# Supplementary material for: Social Isolation Activates Dormant Mammary Tumors, and Modifies Inflammatory and Mitochondrial Metabolic Pathways in the Rat Mammary Gland
Source: Cells. 2023 Mar 21;12(6):961. doi: 10.3390/cells12060961 (PMC10047513; doi:10.3390/cells12060961)
Supplement: Supplementary file 1 [file cells-12-00961-s001.zip › cells-2176292-supplementary.pdf]

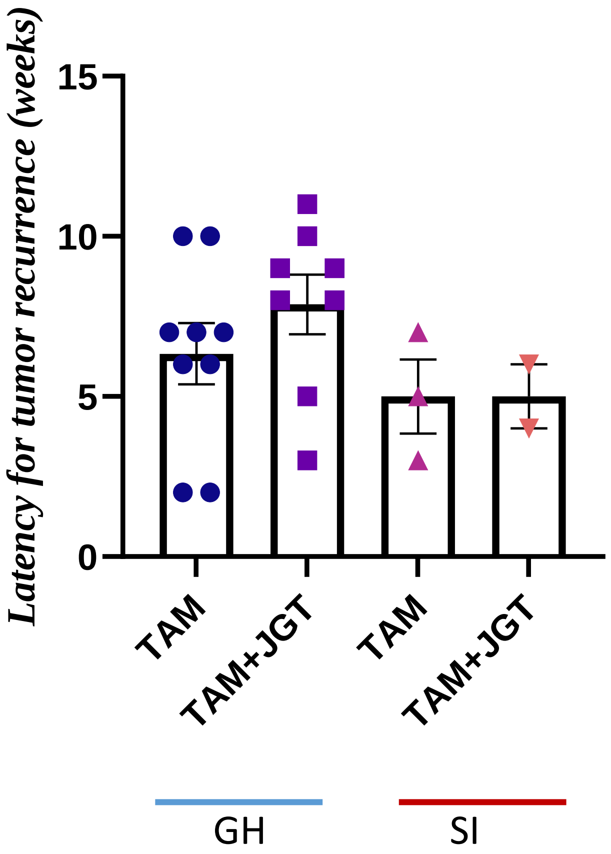

Figure S1. Latency for local tumor recurrence in group-housed (GH) and socially isolated (SI) rats after tamoxifen (TAM) therapy. Rats which received JGT during TAM therapy remained in JGT after TAM was removed.

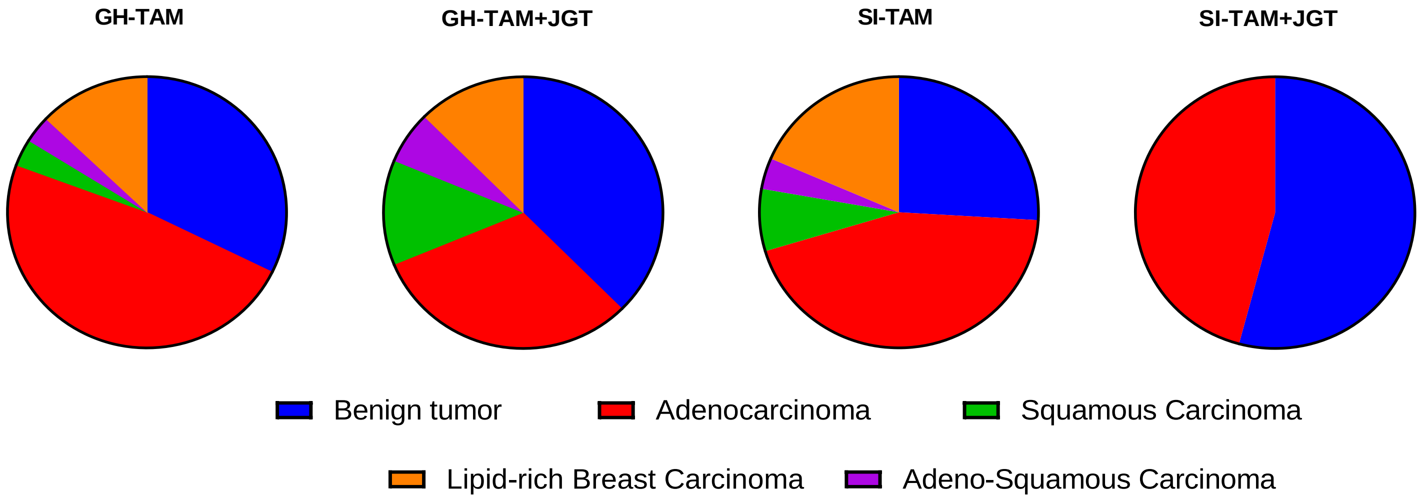

Figure S2. Tumor histopathology of mammary tumors in group-housed (GH) and socially isolated (SI) rats treated with tamoxifen (TAM) or with TAM + JGT. SI did not change the proportion of histopathology compared with GH animals treated with TAM. In GH rats, JGT during TAM decreased (χ^2; P=0.021) the proportion of adenocarcinomas from 48% (15 of 31 tumors) to 31% (5 of 16 tumors) and increased (χ^2; P=0.019) squamous carcinomas from 3% (1 of 31 tumors) to 13% (2 of 16 tumors). In SI rats, JGT increased (χ^2; P<0.001) the proportion of benign tumors from 26% (7 of 27 tumors) to 54% (13 of 24 tumors) and decreased squamous carcinomas (χ^2;P=0.021) from 7% (2 of 27 tumors) to 0% (0 of 24 tumors) and lipid-rich breast carcinomas (χ^2; P<0.001) from 19% (5 of 27 tumors) to 0% (0 of 24 tumors). The proportion of benign tumors (χ^2; P=0.033) in SI rats was 38% (6 of 16 tumors) and in GH rats 54% (13 of 24 tumors), and adenocarcinomas (χ^2; P=0.042) 31% (5 of

16 tumors) in SI rats and 46% (11 of 24 tumors) in GH rats with JGT. However, JGT decreased Squamous Carcinoma (χ^2; P<0.001) from 13% (2 of 16 tumors) to 0% (0 of 24 tumors), Adeno-Squamous Carcinoma (χ^2; P=0.038) from 6% (1 of 16 tumors) to 0% (0 of 24 tumors) and Lipid-rich Breast Carcinoma (χ^2; P<0.001) from 13% (2 of 16 tumors) to 0% (0 of 24 tumors) in SI rats compared to GH also treated with JGT.

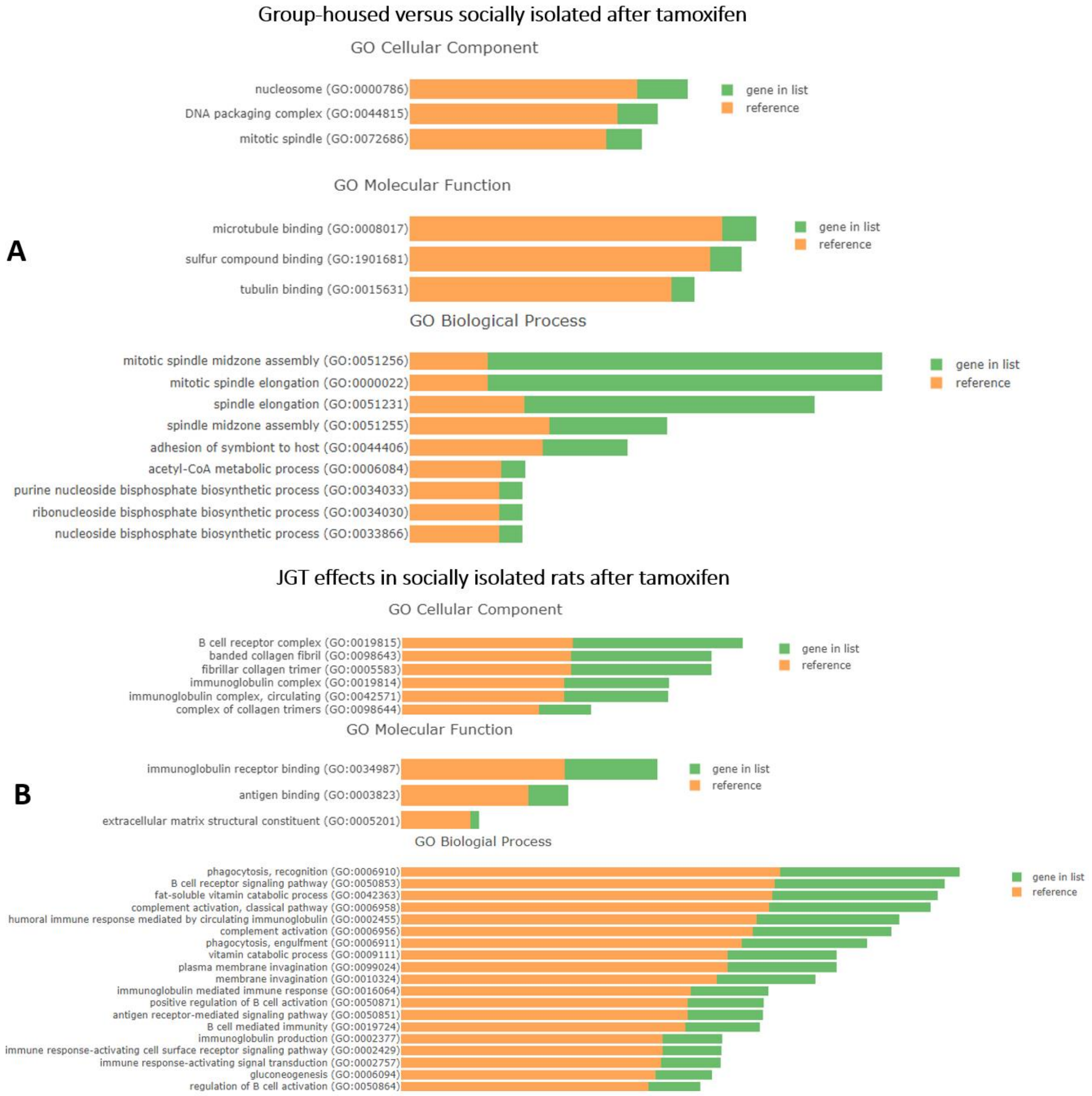

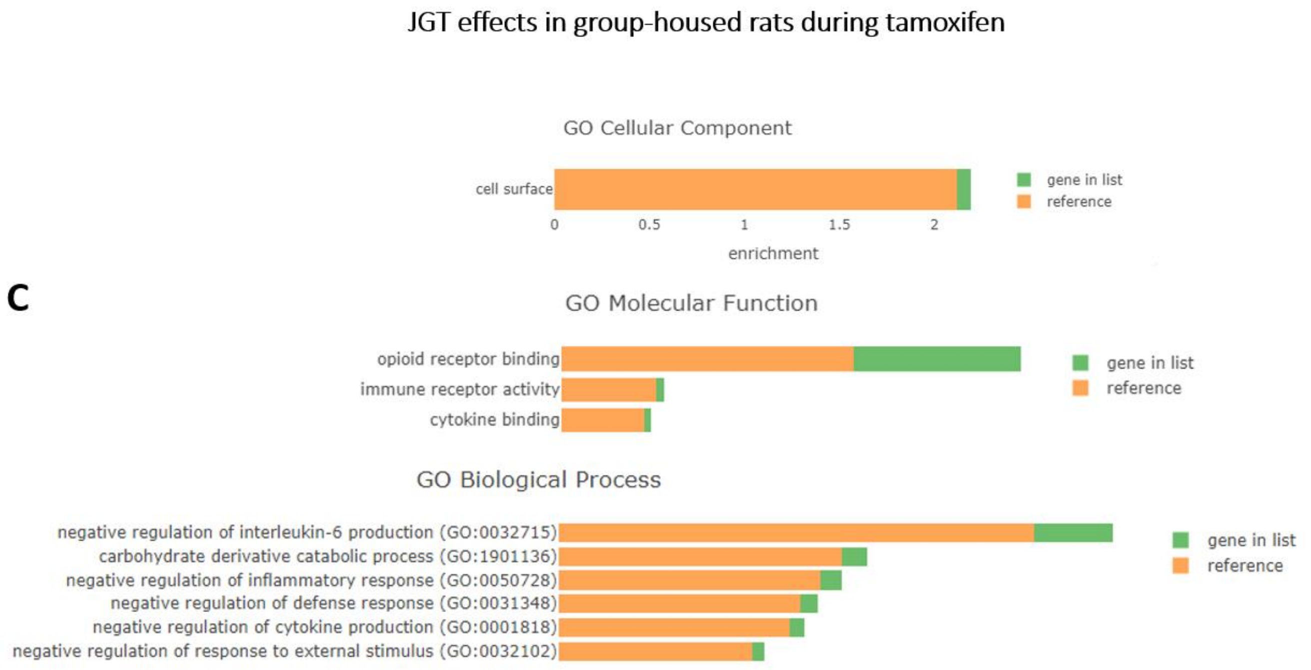

Figure S3. (A). Results of GO analysis between group-housed and socially isolated rats after tamoxifen. **(B**) Results of GO analysis in socially isolated rats between JGT treated and not treated after tamoxifen. **(C)** Results of GO analysis in group-housed rats between JGT treated and not treated during tamoxifen.

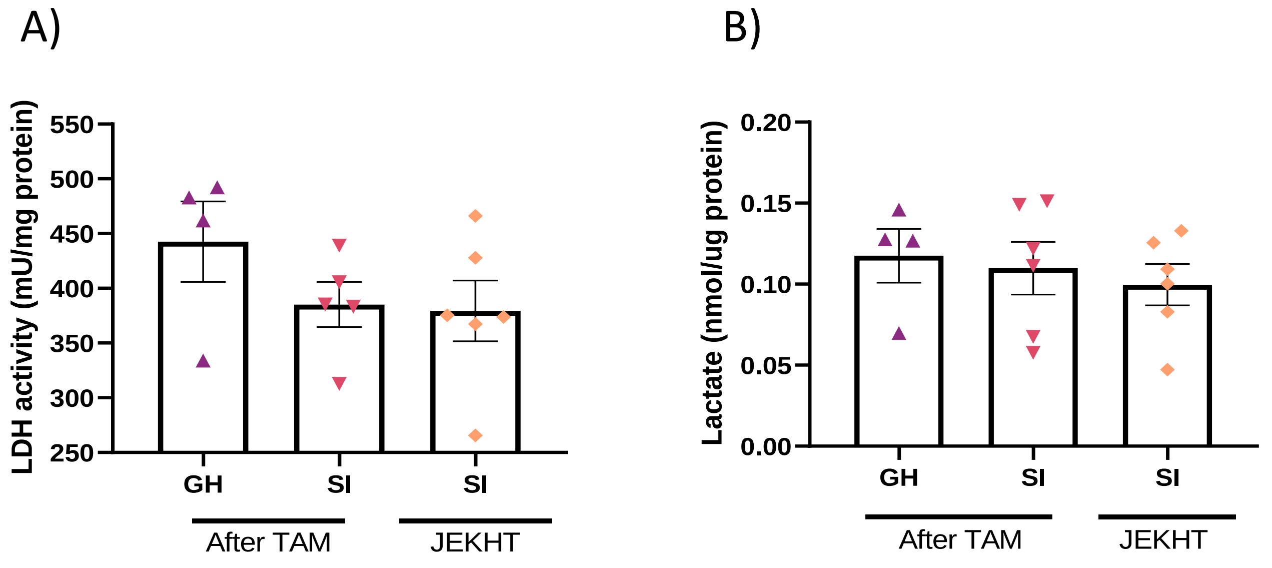

Figure S4. Effect of social isolation (SI) on LDH activity and lactate levels. SI did not alter LDL activity or lactate levels, compared with group-housed (GH) rats or SI rats treated with JGT.

Table S1. JEKHT Composition.

Herb	***Common name***	***Content (g/100 g)***
Paeoniae Radix	Paeonia	10.20
Angelicae Gigantis Radix	Korean angelica root	10.20
Asparagi Tuber	Asparagus cochinchinensis Merr	10.20

Atractylodis Rhizoma Alba	White atractylis	12.29
Rehmanniae Radix Crudus	Rehmannia glutinosa	10.20
Citri Unshii Pericarpium	Dried orange peel	10.20
Anemarrhenae Rhizoma	Anemarrhena	6.14
Phellodendri Cortex	**Phellodendron bark**	6.14
Glycyrrhizae Radix et Rhizoma	Licorice	6.14
Zingiberis Rhizoma Crudus	Ginger	4.05
Liriopis Tuber	Lilyturf	10.20
Zizyphi Fructus	Jujube	4.05

Table S2. Primers used in quantitative real-time PCR.

Gene	Sequence
*Ccl7*_ Forward	5’-CAGAAAGATCACCAGTAGTCGG-3’
*Ccl7*_ Reverse	5’-TCAGGGCTTTGGAGTTGAAG-3’
*Ccl12*_ Forward	5’-TGGCTGGACCAGATTCAGT-3’
*Ccl12*_ Reverse	5’-AGCTCTTTAGCCTCCGAATGT-3’
*Csf2*_ Forward	5’-CTAAATGACATGCGTGCTCTG-3’
*Csf2*_ Reverse	5’-CATTGAGTTTGGTGAGGTTGC-3’
*Csf3r*_ Forward	5’-ATGTCTACACCTACTCTGGAGAG-3’
Csf3r _ Reverse	5’-GATGGTGTAGTTGGTGAGGG-3’
*Cyc1*_Foward	5’-CTCCCATCTACACAGAAGTCTTG-3’
*Cyc1*_Reverse	5’-ACATCTTGAGTCCCATGCG-3’
*Fbp2*_ Forward	5’-GTGGATCTCTTCATGCTGGAC-3’
*Fbp2*_ Reverse	5’-CTGTACATACTCAGCGGTAGC-3’
*Hprt1*_ Forward	5’-GCC CTT GAC TAT AAT GAG CAC T-3’
*Hprt1*_ Reverse	5’-CCG CTG TCT TTT AGG CTT TG-3’
*Idh3g*_ Forward	5’-AACCGTGTGGCTCTAAAGG-3’
Idh3g _ Reverse	5’-TCTAGGCTGGTACGAAGGATG-3’
*Il4r*_ Forward	5’-CTTTGCACCAAGTTCCTGTC-3’
*Il4r*_ Reverse	5’-GTAGAAGTGCGGATGTAGTCAG-3’
*Il6*_ Forward	TTGCCTTCTTGGGACTGATG
*Il6*_ Reverse	GTGGTATCCTCTGTGAAGTCTC
*Il18r1*_ Forward	5’-CATGGTCACGCTTTGGAATTC-3’
*Il18r1*_ Reverse	5’-TTCGTCACAAGCTTCTCAGAG-3’
*Lilrb3*_ Forward	5’-CTCTAGCCCGATTTCAAGTGG-3’
*Lilrb3*_ Reverse	5’-GGTGGGTTTCTGGAGGTTC-3’
*Mcemp1*_ Forward	5’-TTGGCACGGTCAAGAGC-3’
*Mcemp1*_ Reverse	5’-GTGTCCTCTCATTTACTGGGC-3’
*Mdh1*_Foward	5’-CCGGTCAGATTGCATATTCGC-3’
*Mdh1*_Reverse	5’-ACAAGAATGATGGGCTGGTCTT-3’
*Ndufa9*_Foward	5’-CTTCCAATGTCACGTCCTGC-3’
*Ndufa9*_Reverse	5’-CACAACTCCACTGACGGATGA-3’
*Ndufab1*_Foward	5’-GTGTATGACTAAAGTATCAGATGCC-3’

*Ndufab1*_Reverse	5'-TAAGTTCCAGTCAGCAGCCA-3'
*Ndufc1*_Foward	5'-GCTGCTCTTCAACACGGTCA-3'
*Ndufc1*_Reverse	5'-TTCGTTGTGTTGGTGGATGAGATAA-3'
*Rage*_ Forward	GTGAATCCTGCCTCTGAACTC
*Rage*_ Reverse	ACTGTCCCTTTGCCATCAG
*S100a8*_ Forward	GAATCACCATGCCCTCTACAG
*S100a8*_ Reverse	AGCTTTCGGTATTTTTATTCTGCA
*S100a9*_ Forward	CAGTACTCTAGGAAGTATGGACATC
*S100a9*_ Reverse	GGCAAGTCCTTATTCACCATTTC
*Sdhb*_Foward	5'-CAGAGTCGGCCTGCAGTTTC-3'
Sdhb _Reverse	5'-CGGGTCCCATCGGTAAATGG-3'

Table S3. 674 differentially expressed genes between group housed (GH) and socially isolated (SI) rats (after tamoxifen treatment). Criteria for differential expression: p-value less than 0.05, fold change (FC, between GH/SI) greater or equal to 1.5.

Gene symbol	FC	p-value	Gene symbol	FC	p-value
Slc17a8	79.30388	1.22E-38	Vsig8	-28.7788	0.000269
AABR07015081.2	-368.628	2.28E-34	LOC259244	2.004992	0.000299
AABR07015066.1	-117.623	3.43E-32	Sp6	-9.72149	0.000323
AABR07015078.1	-117.623	3.43E-32	Lhx2	-3.9049	0.000332
AABR07015055.1	-117.623	3.43E-32	Kng1	5.263191	0.000341
AABR07015080.2	-117.623	3.43E-32	Slc30a2	-28.3731	0.000348
AABR07063424.1	-117.623	3.43E-32	Pla2g4d	43.32646	0.000448
AABR07015057.1	-145.305	5.36E-31	AC099453.1	-1.57609	0.000448
AABR07000398.1	-149.006	9.09E-23	Cdhr1	12.28983	0.000511
LOC685351	572.7804	1.16E-12	U2	-1.73147	0.00052
5_8S_rRNA	-32.2849	1.84E-12	Tent5b	-6.15929	0.00054
Rn5-8s	-32.2849	1.84E-12	SNORD33	-2.91587	0.000592
Opcml	5.160106	1.01E-07	Alcam	-1.66025	0.000685
Dnph1	-3.00772	2.67E-07	Tnr	-46.5346	0.000685
LOC500035	-128.297	9.67E-07	Pappa2	-7.20356	0.000687
Sv2c	1.989307	1.42E-06	Spdef	-3.94513	0.000708
Padi2	2.243469	2.02E-06	Elovl6	-109.967	0.000716
Acrbp	-1.94391	2.68E-06	Tchh	-34.4762	0.000726
Cacna2d3	2.914457	3.68E-06	LOC103690015	4.018243	0.000726
Moxd1	-39.1366	5.84E-06	Spt1	-22.8632	0.000734
NEWGENE_620180	1.540436	7.23E-06	Gucy1b2	4.007079	0.000746
Lilrb3	3.843915	9.05E-06	SNORD15	-2.33843	0.00077
LOC100364769	-35.0745	1.3E-05	Grem2	2.041266	0.000775
LOC102549542	-9.03755	1.41E-05	Krt31	-2479.08	0.000809
Mlph	-2.38714	4.45E-05	Pnpla3	-3.93041	0.000815

Lrp2	4.395717	5.1E-05	Tnfrsf19	-3.15541	0.000918
Fzd10	-21.8608	5.21E-05	AC116220.1	-2.62553	0.001022
Cds1	-1.89071	7.4E-05	Aldh1a1	-4.71528	0.001049
SNORA73	-8.23493	8.73E-05	Ly6g6e	-2.11423	0.001112
AABR07016992.1	7.183276	0.00012	Cyp2d3	-15.0737	0.001213
Gas2l3	1.962866	0.000135	Elfn1	2.823833	0.001214
Rab3b	2.358233	0.000147	Tph1	5.314266	0.00123
Greb1	3.884361	0.000164	Fer1l6	2.478475	0.00125
Krt71	-179.79	0.000172	Reep1	2.084169	0.001333
Cdca7	-2.00268	0.000175	S100a3	-28.8044	0.001357
Tiparp	1.541562	0.000184	Rspo1	-6.80923	0.001383
Dhcr24	-2.22395	0.000229	Hepacam2	-2.49247	0.001389
AABR07052744.1	-6.11891	0.000236	Mrgprg	4.046062	0.001438
RGD1305184	3.343688	0.000246	Btc	2.307458	0.001485
Aass	-10.0009	0.000248	Tfap2b	-2.17364	0.001549
Tfr2	7.844361	0.001663	Fbxw12	17.73946	0.001627
Smim31	-3.16241	0.001666	Pof1b	-2.19179	0.003597
Trpc4	-8.4178	0.001678	Chn2	1.571287	0.003626
Erbb4	-1.94462	0.001712	Azgp1	-9.19192	0.003705
Tkt	-2.96685	0.001732	Dhrs7	2.329446	0.003824
Fbn2	-2.29698	0.001777	LOC108348130	-2.85614	0.003832
Actbl2	-68.5991	0.001785	Mlana	-38.7733	0.003843
Cbln1	4.004488	0.001933	Pank1	-2.42833	0.003988
LOC102552398	-9.79152	0.002006	Col11a1	-3.89355	0.004084
Grm8	-7.28821	0.002064	Sptssb	-5.78772	0.004148
Fbp2	-2.15318	0.00209	5S_rRNA	-2.67435	0.004199
Gpr155	1.53971	0.002119	Slc6a14	-3.59435	0.004334
Hpx	-5.5292	0.002162	Pinlyp	-5.68046	0.004414
Bhlha15	-5.02733	0.002181	Rnd1	1.93027	0.004507
Siglec10	2.751806	0.00221	Krt75	-26.4617	0.004528
Ace2	-1.87511	0.002292	Acy1	-1.61162	0.004618
Itih1	2.257192	0.00234	Atp6v1c2	-6.75079	0.004859
Dapk1	1.622869	0.00243	Apobec2	-7.94145	0.004931
Adra2c	3.470089	0.002449	Esrrb	2.404293	0.005132
Pcdh17	1.62375	0.002485	Igfbp4	1.764683	0.005166
Gm25848	-1.83198	0.002485	Il31ra	2.151696	0.005286
Angptl8	-3.22101	0.002504	Snord49a	-4.57275	0.005361
Il4r	1.918057	0.002644	Gm25776	-2.07953	0.005439
Cdt1	-1.86453	0.002656	Mup5	1.890911	0.005489
Plppr1	-48.9771	0.002695	Nt5dc2	-3.9569	0.00581
LOC100912373	-5.2002	0.002705	Ak6	-1.72961	0.005911
Krt86	-524.846	0.002765	Clip4	1.676428	0.005975
Acly	-2.66469	0.002774	Scd	-6.10194	0.005976
Calhm4	-9.55469	0.002942	LOC103690190	-4.45867	0.006029
Aldh3a1	2.127032	0.003141	AABR07018050.1	11.25982	0.00607
Rasgef1a	3.826775	0.003148	Tas1r2	-1.50081	0.006162

Sidt1	-2.15594	0.00319	Adh7	1.778862	0.006184
Snora9	-2.34503	0.003275	Calca	-17.2983	0.006193
Thop1	-1.67526	0.003337	Krt81	-550.94	0.006222
Slc6a20	-2.50185	0.003365	Thrsp	-2.59077	0.006227
Krt28	-32.2254	0.003366	Usp2	-1.99112	0.006483
Nabp1	1.879507	0.003374	Sult2b1	-2.25032	0.006538
Acaca	-3.71176	0.003391	LOC100361025	2.281635	0.006638
AABR07059372.1	7.07073	0.003418	Pxdc1	1.646807	0.006653
Rsph10b	2.089258	0.00351	Ehhadh	-2.49499	0.006712
Rnd2	1.513645	0.003553	Tbc1d30	-2.76232	0.006789
LOC100909548	-2.24924	0.006991	Terc	-3.28343	0.006877
Dnah5	2.130485	0.006999	Sox15	-2.17272	0.009644
Olfm4	-32.1495	0.007003	Thbs1	2.138383	0.009681
Gcgr	-4.14473	0.007098	Ifi44l	1.505248	0.009919
Serpina3m	-15.839	0.0071	Tlcd4	-1.65114	0.009931
Tecr	-1.80155	0.00718	Cebpzos	-1.67229	0.010008
Echdc1	-1.51694	0.007182	Lep	2.369133	0.010053
Ifit3	2.013434	0.007192	Espn	2.406937	0.010116
Adh6	-9.12276	0.007195	Star	-4.56265	0.010172
Phlda1	-5.21344	0.007332	Kyat3	1.773076	0.01024
AC114512.2	-2.01466	0.007341	Shisa4	1.5456	0.010265
Fras1	-3.05014	0.007389	Klhl40	-13.2405	0.010398
LOC680322	-3.85231	0.007436	Slc22a20	12.55595	0.010434
Acta1	-4.44203	0.00744	AABR07034648.1	-8.65396	0.010497
Unc80	-4.36423	0.007449	Wfdc18	12.16712	0.010564
Msx2	-3.4508	0.007499	Siglec5	2.652079	0.010779
AABR07010460.1	-1.59828	0.007523	Klk1	-79.84	0.010806
Oxtr	2.109614	0.007568	Rnf222	-29.4124	0.011039
Rxfp1	1.866461	0.007621	LOC685849	-2.21602	0.011042
Chrm2	8.471442	0.007661	Tecta	-10.5818	0.011183
Dlg2	1.878614	0.007717	Idi1	-1.51246	0.011266
Irx3	-1.92898	0.007728	LOC102550080	12.1034	0.011316
Fut2	-2.75336	0.007757	Plpp4	3.884482	0.011335
Krt32	-376.531	0.007835	Isoc2b	-1.71889	0.011446
LOC108348096	-4.24213	0.007912	Mns1	-3.32175	0.011539
Il18r1	1.582542	0.007941	Tnmd	2.138956	0.011568
Csap1	-4.92381	0.007946	hist1h2ail2	-3.73398	0.011573
Map3k13	-1.69072	0.007998	Rps6ka6	-2.48606	0.011628
Dhrs7l1	2.10889	0.008199	Pde4c	-2.18707	0.011701
Camp	-12.388	0.008212	Angpt4	2.381877	0.011708
Mpc1	-2.61065	0.00823	Ptprh	-12.5643	0.011768
Tdrd5	2.609804	0.008577	Crlf1	1.862618	0.011772
Dbi	-1.6581	0.00877	Cldn10	-4.40689	0.01178
RGD1309534	-2.59711	0.008922	Hist3h2ba	-1.79274	0.011813
Krtap3-1	-317.531	0.009087	AABR07042999.1	-1.6113	0.011847
Vwa2	-3.2326	0.009125	LOC103689983	-1.67576	0.011897

AABR07058706.2	-3.76044	0.009378	Plk5	-4.0009	0.011923
Spon1	1.574356	0.009491	H2bc1	-3.66221	0.011942
Rtel1	-1.61163	0.009546	Krt39	-33.6788	0.012157
AABR07007068.1	1.925545	0.009584	AABR07002792.2	-2.16061	0.012228
Spc25	-4.38867	0.009593	Serpina12	-5.24624	0.012312
Enpp2	1.816996	0.012562	Hist1h2ail1	-4.04324	0.012558
Kif26b	-3.13455	0.01264	Kcna2	-3.01875	0.015517
Shroom1	-1.77128	0.012648	RGD1563349	2.160505	0.015877
RGD1304624	-1.62599	0.012769	Gria1	2.257649	0.015959
Cpa1	1.930223	0.012829	Dusp4	-1.72438	0.016056
Slc25a1	-2.10145	0.013004	Krt1	6.079695	0.016227
Fbp1	-13.7005	0.01303	Kif2c	-2.71151	0.016267
Cst6	-1.85476	0.013054	Mup4	1.874944	0.016283
P2ry6	1.653896	0.013104	Gpsm2	-1.79902	0.016287
Med22	1.586809	0.013312	Peg3	1.784543	0.0165
LOC684773	-3.91509	0.013327	St8sia5	-4.97575	0.016868
SNORD22	-1.72945	0.013365	Cacng4	2.172911	0.016943
Hist2h3c2	-4.43952	0.013444	Unc5cl	2.161754	0.01696
LOC102548682	-4.43952	0.013444	Grb7	-1.76917	0.017073
Gzmb	-6.31159	0.013474	Txlnb	-5.53177	0.017138
Gnmt	-8.17281	0.01349	Capn6	-3.10813	0.017207
Serhl2	-2.80095	0.013498	Gas6	1.824566	0.017277
Hist1h2an	-4.22956	0.0135	AABR07065781.1	5.172828	0.017376
Trpv1	1.823543	0.013626	LOC100365839	-1.53371	0.01739
Fam25a	-13.5827	0.01387	Phf6	-2.34249	0.017405
LOC103693563	-2.13741	0.013943	7SK	6.703279	0.017548
Cnfn	-30.9979	0.014011	Pla2g7	3.312676	0.017609
Cers3	-14.0045	0.014017	Ppef2	2.297368	0.017613
Foxred2	1.529318	0.014086	AABR07006624.1	6.709963	0.017619
LOC100910554	-3.94895	0.014102	Lrrc15	-4.21177	0.0178
Spink5	-13.28	0.014251	H2bc12	-3.3144	0.017952
Nrtn	-2.81943	0.014284	Hist1h2ak	-3.53504	0.018083
Arg2	-4.05652	0.014303	Hrh3	-3.82523	0.018115
Hs3st6	-2.04569	0.014438	Rarres2	1.622553	0.018439
Lilrb2	1.719465	0.014509	Grb14	2.062153	0.018457
Krt79	2.337781	0.014624	Evpl	-1.90264	0.018584
H1f5	-3.91049	0.014721	LOC684762	-3.39063	0.018611
Mlf1	-4.51031	0.014768	Fam78b	2.154201	0.018996
AABR07035412.2	1.6186	0.014823	Unc5b	1.74435	0.019022
Tectb	-6.02818	0.014842	Nhp2	-1.5828	0.019041
Ptges3l	-1.54666	0.015262	Herc6	1.577565	0.019061
Ankle1	-5.20293	0.015295	Trem3	-3.45868	0.019192
Padi3	-88.3393	0.01534	LOC299282	1.955825	0.019235
Rnu3a	-1.60953	0.015347	AABR07064613.2	1.528872	0.019294
AC141152.2	-1.79115	0.015363	Foxa1	-1.93117	0.019378
Psors1c2	-82.6275	0.015397	Rab9b	2.360066	0.019428

LOC685067	1.929572	0.019447	Itpka	-2.05572	0.019429
Ngp	-15.5709	0.019508	Bcan	2.998534	0.022778
Ppp1r3a	-13.0314	0.019546	Arhgap40	-6.1003	0.022793
Zfp296	-2.07263	0.019694	Ccnf	-2.89856	0.022809
Chst8	2.926441	0.019722	Cdc7	-1.52561	0.022895
Xbp1	-2.14686	0.019889	Hist1h2af	-2.93974	0.023018
Mgme1	-1.63981	0.019973	Sncg	2.056211	0.023117
Pcnx2	3.235296	0.019983	Gm22620	-1.86299	0.023162
U1	20.93408	0.020188	Apobec3	-3.43509	0.023228
Gchfr	-2.37529	0.020208	Kif18b	-3.01917	0.023357
Atp6v0d2	6.170891	0.0203	AABR07052585.2	-4.9106	0.023386
LOC103690070	-3.83799	0.020304	AABR07067827.4	-1.67162	0.023691
Slc22a7	-5.31317	0.020347	Acer2	1.747722	0.023711
Gys2	-4.46428	0.020543	RGD1562011	5.988699	0.023814
AABR07016856.1	2.852569	0.020555	Cep72	-3.26325	0.024011
Pdhb	-1.6406	0.020613	Lefty1	-7.32163	0.024073
Retn	-1.71106	0.020665	Slc22a3	-2.55147	0.02428
Klf5	-1.5607	0.020686	Cenpf	-3.11407	0.024326
Man1a1	1.52875	0.020688	AABR07028795.2	1.950005	0.024442
Lgr6	-1.85011	0.020695	Stab1	1.606401	0.024532
Slco2b1	1.635876	0.021171	Rspo3	1.75888	0.024658
Slc5a6	-1.5829	0.02152	Glyctk	-1.61098	0.024701
LOC688778	-2.99327	0.021553	Corin	-2.83367	0.024778
LOC100359668	11.2625	0.02175	Daglb	2.011107	0.024847
Grin1	-2.15187	0.021756	Gcat	-1.68718	0.024881
Kif21a	-2.12276	0.021758	F2rl3	1.501428	0.024883
Slit1	-7.18169	0.021765	Cdca3	-3.7353	0.024926
Ogdhl	2.166264	0.021908	Lynx1	-3.0091	0.025041
AABR07027811.2	-3.50158	0.02192	Htra4	1.725926	0.025098
Dennd2d	-2.02067	0.02192	Il22ra1	-4.9327	0.025205
SNORA74	-1.58644	0.02196	LOC100909700	-9.49221	0.025269
Tk1	-1.95135	0.021999	H2ac1	-2.54229	0.025355
Hist1h2ah	-2.74146	0.022175	Wscd2	2.230929	0.025393
Tnni2	-5.55663	0.022244	Smim5	-2.00671	0.025469
Tlcd3b	-3.05135	0.022431	Togaram2	2.374625	0.025527
AABR07067401.1	-2.51215	0.022477	Msrb1	-1.66015	0.025604
Nusap1	-3.78029	0.022544	Pdk1	-1.9231	0.025898
Kl	5.359191	0.022599	Lgi2	-12.3836	0.025913
Kcnab1	1.709499	0.022671	Prom1	-2.00641	0.025944
AABR07065789.3	4.643754	0.022686	Zfp428	-1.60974	0.025977
Gipc2	1.64353	0.022757	Lgals7	-2.45938	0.026271
Gm23650	-3.60272	0.026399	Kcnn4	-3.33359	0.026278
Dctpp1	-2.10934	0.02644	Snord67	-1.99375	0.031084
Celf6	-5.80911	0.026737	Entpd3	-2.92856	0.031101
Kcnq4	1.903607	0.026806	Slc4a1	-3.04422	0.031232
Akr1c19	1.819875	0.027022	Gm23202	-3.50096	0.031361

Gpr88	1.918996	0.027089	Cd99l2	1.501151	0.03137
Acot1	-2.60389	0.027139	Trim45	-2.06619	0.031421
Ampd1	-5.66958	0.027177	LOC100910798	-5.60894	0.031576
Mdh1	-1.52418	0.027269	Gsta4	-1.94496	0.031637
Mrpl38	-1.56733	0.027691	Cldn1	-1.64281	0.031699
Sema3f	1.554724	0.027851	Ppp1r27	-6.20794	0.031737
P2ry14	1.692231	0.027879	Prps2	1.79276	0.031782
U5	-2.10804	0.028009	Mmp8	-7.06757	0.031828
Ppp1r1b	-2.44874	0.028024	Tmem110	-1.55092	0.032012
Adcy5	1.622943	0.028052	U12	-1.7404	0.032078
Igfals	2.201949	0.028063	Eno3	-2.68374	0.032114
Muc1	-3.12318	0.028065	Mpc2	-1.62593	0.032243
Plekhg4	-3.38727	0.02809	Cks2	-3.13746	0.032246
Clca4l	1.826019	0.028117	Dsg1	-14.1752	0.03235
Ckap2	-3.05592	0.028132	Kcng2	-2.59758	0.032508
Clca4	-2.89153	0.028223	LOC102549173	-3.05409	0.032646
Nupr1	-2.22112	0.028231	Haus8	-1.57726	0.032682
Kcnj11	-2.56977	0.02832	Gnl2	-1.7881	0.032882
AABR07062138.2	1.723747	0.028338	Cox6a2	-6.78018	0.032887
Myl1	-4.33804	0.028428	Wnt2b	1.650499	0.032951
L1td1	-2.09383	0.028684	Tube1	-1.52681	0.033121
Pus7	-1.57924	0.028877	Fosl2	1.510711	0.033345
Gm25835	-1.83944	0.029011	H4f3	-2.41758	0.033346
Tpx2	-2.99451	0.029031	Nkx1-2	-8.97156	0.033388
Nrap	-8.54404	0.029379	Dapl1	-6.07836	0.033448
Glrb	-3.6658	0.029379	AABR07026473.1	3.01772	0.033675
Slc37a2	1.543687	0.029391	Mest	2.335782	0.033678
AABR07021759.1	1.716391	0.029888	AABR07051324.1	-2.075	0.03373
Cmtm8	-1.63504	0.02996	AABR07051325.1	-2.075	0.03373
AABR07038886.1	-2.11766	0.029973	AABR07056026.1	3.820345	0.033878
AABR07045400.1	7.884657	0.030199	Padi4	-2.18945	0.033906
Rassf6	-1.83978	0.030611	Map1a	2.199008	0.033928
Hist1h2bo	-2.79997	0.030705	Slco1a1	2.934699	0.03396
Cxcr2	1.738165	0.030768	Tgm6	-27.4782	0.033972
Pde1c	1.528253	0.030839	Sptb	-2.66201	0.034037
Ttc25	-2.00946	0.031047	Ace	1.537502	0.03404
Smtnl1	-7.19945	0.034249	Ube2t	-4.11767	0.034247
Smim38	3.535139	0.034265	Gng13	-1.79706	0.037158
Rnu11	-1.69257	0.03427	LOC685203	-2.90639	0.037375
AABR07061187.1	-1.69257	0.03427	Fam83f	-1.70083	0.037478
AABR07067138.1	-1.69257	0.03427	Slc7a15	1.874635	0.037489
Nrxn1	1.828096	0.034277	Bmp3	1.863413	0.037582
Ca5b	-2.57169	0.034302	LOC100911692	-2.01956	0.037763
Kif20b	-3.05992	0.034346	Chac1	-4.54895	0.037793
Mycbpap	1.609132	0.034388	Pla2g4e	-7.3556	0.03783
AABR07048482.1	3.529107	0.034641	Kif4a	-3.24505	0.037963

Tent5c	-2.05012	0.034782	Pdgfa	-1.83351	0.03797
Mb	-5.1658	0.034796	Slc12a2	-1.62357	0.03798
Bmp4	1.638228	0.034799	Slc43a3	1.80812	0.038006
AABR07061328.1	-2.2003	0.034839	LOC100912564	-2.28149	0.038013
Cdc25b	-1.502	0.034846	Snord8	-1.78533	0.038098
Ptgfr	1.791628	0.034981	Ccl21	1.541944	0.038154
LOC100911660	-2.20766	0.034987	Hist1h3b	-2.71364	0.038246
Fam83d	-4.25396	0.034998	Ms4a4e	1.535695	0.038348
Prob1	1.607431	0.035151	Clca2	-2.20838	0.038362
Sstr4	10.23652	0.035212	Dnase1l3	1.673935	0.038417
AY172581.15	-2.6758	0.035321	Hist1h2bl	-3.05987	0.038738
LOC691661	-6.69221	0.035343	LOC100910851	-6.8367	0.038781
Proz	-3.32693	0.035356	AABR07011682.1	-2.12389	0.038823
Gbp4	1.760772	0.035388	Kif22	-2.77877	0.038921
Ccn3	1.643584	0.03557	Calml3	-1.95091	0.039087
NEWGENE_1308171	1.554294	0.035623	Gnas	-3.1743	0.039118
Fads2l1	-10.7736	0.035625	Rtkn	-2.1581	0.03912
Lvrn	1.825461	0.035659	RragB	5.672268	0.039242
Ckap2l	-3.41459	0.035711	Slc18a2	1.586547	0.039281
Racgap1	-3.12484	0.035775	Ccl24	1.6455	0.039305
LOC102549061	-2.7018	0.036015	Gpam	-1.9859	0.039459
Cd300lf	1.739293	0.036268	Tmc5	-4.18241	0.039474
Necab1	-1.70475	0.03631	Sap30	-2.19674	0.039538
Pirb	1.500969	0.036411	Xirp2	-5.70265	0.039617
AABR07061825.2	2.493463	0.036554	Chrnb4	-3.26145	0.03963
C1rl	1.656712	0.03657	Mcc	-1.94725	0.039697
Kcnc3	4.291763	0.036587	Ckmt1	-2.84219	0.039715
Prxl2a	-1.50143	0.036623	Ptgds	2.085916	0.040242
Me1	-3.04159	0.037004	Rpl3l	-6.70885	0.040324
Otogl	3.980157	0.037035	Krt18	-2.02661	0.040456
LOC691670	-7.36862	0.037128	Tex13b	-4.79722	0.040475
SNORA70	-1.67714	0.04055	Cxcl10	1.984239	0.04054
Slc6a19	-3.91557	0.040694	Cbx2	-2.4208	0.044821
Ly6g6d	-3.81551	0.040767	Pole2	-2.22577	0.044836
AABR07005779.3	12.42652	0.040955	Nxnl1	-3.63063	0.045163
Pcdhb2	1.812037	0.04107	Samd9	1.526656	0.045224
Gulo	-3.49052	0.041439	Kif11	-3.13018	0.045347
Melk	-3.7194	0.041461	Olr836	3.439198	0.045457
Neb	-3.25931	0.041603	Nupr2	-1.53159	0.045644
Pltp	1.534502	0.041675	Dsc1	-15.7234	0.045659
LOC499796	3.086731	0.041706	Plxna4	1.56151	0.045666
Sema5b	2.07748	0.041721	Gucy2g	-7.41785	0.045731
E2f8	-4.13783	0.041999	Ndc80	-2.70656	0.045771
F12	-9.96934	0.042025	Kank4	-2.28423	0.045809
Nceh1	1.504043	0.042088	Six4	-1.83139	0.045889
Abo3	-3.0579	0.042107	Klhl13	1.572787	0.045929

Vil1	-6.38048	0.042167	U3	-1.60439	0.046029
Myo1d	1.518193	0.042238	Sfn	-2.16541	0.046037
Prmt1	-1.56714	0.042508	Fasn	-2.25918	0.046143
Tmprss9	1.83767	0.042776	Lbhd2	-11.2468	0.04631
Lsamp	1.714392	0.042806	Ipcef1	1.578077	0.046691
AABR07069282.1	-3.38984	0.042838	Lrrc26	-2.39529	0.046789
Cxcr4	-1.97065	0.042862	Ovol1	-2.03811	0.046815
Orc1	-2.34303	0.042962	Mmp7	5.476188	0.046872
Adssl1	-1.7445	0.043076	H2ax	-1.98574	0.046962
Dsc2	-1.5526	0.043114	Metazoa_SRP	-1.91429	0.047005
Acer1	-6.56461	0.043296	Plch1	-3.83853	0.047122
Cblc	-2.17455	0.043579	Fgg	1.895283	0.047153
Pc	-1.96362	0.043581	AABR07072025.1	-1.64421	0.047165
Mastl	-2.26747	0.04365	Spc24	-3.07856	0.047174
Rps20	-2.48031	0.04379	Atp2b2	4.721358	0.047357
LOC684797	-2.64054	0.043899	AABR07068279.2	-1.53256	0.047364
Mx2	2.748558	0.043916	Aurkb	-2.75485	0.047463
Mnd1	-2.00052	0.0443	Knstrn	-2.82949	0.047605
LOC100912489	-3.05286	0.044354	Elane	-6.68556	0.047887
Ncald	-1.60582	0.044461	Tgm5	-3.25361	0.047919
Cdkn1c	1.618948	0.044471	Lum	-1.54829	0.047929
Prc1	-2.84587	0.044505	Cdh26	-15.276	0.047932
AABR07025140.1	1.621479	0.044511	Actn2	-5.87315	0.04799
H3c1	-2.91669	0.044611	Creg1	-1.53957	0.048023
Pcdhga2	1.643964	0.044712	Klhl41	-3.63853	0.048024
Slfn2	1.564167	0.044817	Oacyl	-3.85289	0.048264
Rragd	-2.41806	0.048322	AC098008.2	5.811842	0.048293
Troap	-3.46838	0.048331			
Haus4	-1.60029	0.048365			
Tekt5	-11.5881	0.048378			
Hsf2bp	-1.62863	0.048652			
Plpp3	1.586462	0.048657			
Vash1	1.519719	0.048665			
H2ac20	-1.81866	0.048927			
Orm1	-3.7738	0.049007			
Mybpc2	-3.34355	0.049263			
Abcc8	-3.8377	0.049293			
Wfdc21	4.286564	0.049418			
Klhl31	-3.8102	0.049418			
Kif23	-2.71563	0.04943			
Pclaf	-3.47394	0.049453			
Sgo2	-2.6087	0.049759			
Laptm4b	-1.72481	0.049922			
Snord83b	-1.59779	0.049968			
Fxyd2	1.576634	0.049974			

Table S4. 349 differentially expressed genes between socially isolated rats (SI) treated with JGT and SI rats not treated with JGT. Criteria for differential expression: p-value less than 0.05, fold change (FC, between SI+JGT/SI) greater or equal to 1.5.

Gene symbol	FC	p-value	Gene symbol	FC	p-value
Clca2	-2.25432	8.78E-10	AABR07065789.3	-7.34243	0.000359
Col3a1	2.389134	8.98E-06	AABR07051716.2	-33.5311	0.000469
NEWGENE_621351	2.011811	1.23E-05	Adamts18	2.497181	0.000571
Knop1	40.01095	1.40E-05	Col5a2	1.637323	0.000597
Csap1	3.948093	2.54E-05	LOC102549542	3.385151	0.000612
Col1a1	2.405508	3.92E-05	LOC100912373	2.545694	0.000621
AABR07001512.1	-2.15368	4.73E-05	Cpxm1	1.763825	0.000667
Scd2	1.647719	6.64E-05	Clec11a	1.819085	0.000713
AABR07030366.1	2.346306	7.53E-05	Clybl	1.509032	0.000781
Lum	2.232773	8.10E-05	Kl	-8.67375	0.000822
C1qtnf6	1.708285	9.00E-05	Sell	-3.23812	0.000824
Rnasel	1.615594	0.000107	LOC100909620	3.201776	0.000843
Mrap	1.592053	0.00011	Angptl8	2.868158	0.00086
Tmem150c	1.633879	0.000141	Wfdc1	1.623051	0.000918
Pinlyp	6.591762	0.000143	AABR07065766.1	-24.8247	0.000926
AABR07051731.1	-6.77661	0.000144	AABR07065781.1	-6.34252	0.000942
Plppr4	2.229182	0.000145	Crabp1	1.511759	0.000967
Usp44	-5.36565	0.00018	Cwh43	2.954368	0.000988
Cfap77	2.469942	0.000197	Angptl4	-1.85571	0.000995
AABR07043892.1	4.068612	0.000217	AABR07051533.2	-7.495	0.001016
Spef2	2.848518	0.000226	Star	1.947904	0.001119
Postn	2.049339	0.000239	Gp2	3.073899	0.001171
Otos	1.888223	0.000272	Pcdh10	2.481903	0.001239
Cpz	1.756047	0.000275	Rprm	1.9655	0.001296
Msx1	1.718987	0.000289	Rbp4	1.500003	0.001372
Slc6a14	2.707085	0.000306	AABR07013111.1	-4.81516	0.001375
Dpt	1.962097	0.000307	Ca5b	1.601149	0.00145
LOC102551265	28.79062	0.00157	AC123427.1	4.468333	0.001521
Atp6v0d2	-10.896	0.001583	Thy1	1.535203	0.004115
AABR07065789.2	-36.4929	0.001634	Lefty1	2.510807	0.004211
AABR07060980.1	-15.5367	0.001684	Rasd1	1.599866	0.004355
LOC100911837	-1.81433	0.001765	Hephl1	-22.1375	0.004526
AABR07039461.1	-17.0921	0.001858	Mfap2	1.680429	0.004586
Kcnj11	2.579033	0.00189	AABR07032520.1	-3.4932	0.004592
Cyp26b1	-1.7394	0.002349	Pnpla3	4.166413	0.00467
Shc4	1.719198	0.002379	AABR07051611.1	-3.45824	0.004677
Glycam1	-31.1099	0.002458	Wfdc18	-10.142	0.004733
Abcc8	2.397791	0.002541	Scml4	-2.36938	0.004792
Tnfrsf14	-3.44151	0.002552	AABR07010620.1	-1.63034	0.004887
Tfcp2l1	-1.54093	0.002589	AABR07050149.1	-4.17403	0.004976
Gpam	1.656396	0.002678	Acaca	1.979688	0.004979

AABR07015687.1	-1.57991	0.002801	AABR07065780.1	-7.11367	0.005193
Rps20	4.179014	0.002885	AABR07043098.1	3.310129	0.005263
AABR07065883.1	-12.7852	0.002979	Rpl3l	5.353273	0.005397
AABR07065827.1	-7.45008	0.003076	Tectb	3.234953	0.005576
AABR07051592.2	-5.76427	0.003076	Aldh1l2	1.751373	0.005584
Cfi	-1.96223	0.003183	AABR07051733.2	-27.4874	0.005709
Adam12	1.633216	0.003192	Pc	1.521304	0.005759
Ppic	1.641442	0.003317	AABR07056026.1	-6.4434	0.005865
AABR07051684.1	-7.5776	0.003335	AABR07051562.1	-5.73145	0.005881
U2	-4.81151	0.003336	Hmgcs2	-1.89908	0.005899
Armcx2	1.658916	0.003587	Gpd1	1.520617	0.006026
LOC102553715	1.837343	0.003719	Cyfip2	-1.74508	0.006174
Mmp7	-11.5086	0.003773	Abca17	3.468095	0.006411
Atp2b2	-7.352	0.003927	AABR07051583.1	-9.96362	0.006412
Ly6al	4.837333	0.006427	Sdcbp2	-3.86649	0.006417
Tbx21	-1.85868	0.006564	Krt1	-5.24657	0.010011
Tspan8	1.593866	0.006625	LOC103694381	-3.12761	0.010206
Kif1a	-1.75729	0.006898	Prr29	-1.5877	0.010249
Il17b	1.634722	0.00703	AABR07059372.1	-6.54928	0.010379
Mpc1	1.541078	0.007073	Htr5b	2.612351	0.010424
Tmprss11a	-44.3527	0.007152	Cxcl6	-4.20841	0.010483
Rad54b	2.17891	0.007171	AABR07060952.1	-36.6656	0.010606
Tnni2	3.250586	0.007538	Srpx2	1.652432	0.010666
Itm2a	1.869334	0.007544	Ttc25	1.518165	0.010774
Myl1	2.836029	0.007584	LOC102554913	-5.70128	0.010788
AABR07017635.2	-4.88706	0.007671	Xirp2	4.57795	0.011234
Ces4a	1.571908	0.007719	Myoz1	3.001816	0.011368
Pdk4	-2.16443	0.00775	Ces2h	1.632033	0.011464
Rims2	1.521329	0.007869	Cmahp	-1.57256	0.011763
Elovl6	28.21849	0.007931	Tcf7	-1.79417	0.011803
LOC103689971	-5.57302	0.008068	Aspn	1.611665	0.011972
LOC100910790	1.767126	0.00818	Retn	1.593773	0.012074
AABR07060487.1	1.77589	0.008288	RGD1311744	1.570227	0.012285
Npw	1.903962	0.008361	LOC103693564	2.891993	0.012328
AABR07065776.3	-4.06943	0.008467	Ldlr	1.601598	0.012351
C1qtnf5	1.583248	0.008488	Golga7b	-6.52847	0.012482
Ptprv	1.853264	0.00862	AABR07060872.1	-3.5949	0.01251
Gpr88	-1.54321	0.008714	Ccl3	-2.64989	0.012635
Smim31	2.581809	0.008872	Tlcd3b	1.658066	0.012678
AABR07065772.3	-3.76179	0.009516	AABR07065811.1	-4.21373	0.013526
Aox3	1.53753	0.009672	Ccr7	-2.5223	0.013528
Tfr2	-4.51153	0.009873	Abcg8	-2.83428	0.013605
Tnfrsf19	1.554464	0.013688	Spp1	-10.7022	0.013664
AABR07065768.3	-7.3809	0.013705	Insig1	1.684804	0.0183
AABR07049768.1	11.12167	0.014139	Mchr1	-4.61113	0.01877
Cox6b2	1.503078	0.01419	Slc28a3	-5.75755	0.018948

Mybpc2	2.418753	0.014337	Pimreg	2.572513	0.019215
Fbxw12	-5.34693	0.01459	Serpine3	-8.29518	0.019848
AABR07065815.2	-6.07035	0.014599	Lck	-1.70119	0.019899
Jchain	-3.10683	0.014964	LOC100912566	-8.53612	0.020168
Fut2	1.572227	0.015011	Slitrk6	1.734004	0.020438
Metazoa_SRP	2.712742	0.015425	Elavl4	-11.2136	0.020523
Izumo4	1.8675	0.015977	Krt27	18.03364	0.020549
Ppp1r3a	5.208805	0.015999	AABR07029023.1	-1.84911	0.020598
AABR07051716.1	-10.8061	0.016052	AABR07027450.1	-7.04194	0.020613
Il21r	-2.2869	0.016299	LOC102554659	1.833952	0.020724
LOC108351589	-1.86478	0.016551	Il1rapl1	2.175415	0.021011
Slc34a2	-4.71601	0.016576	Slamf6	-2.64047	0.021057
Chrdl2	-8.81148	0.016947	Mmp12	-2.30637	0.021229
Corin	1.995926	0.016979	Gpr39	-1.90451	0.021343
Blk	-1.80015	0.017349	Smoc2	1.610276	0.021444
Ighm	-2.65351	0.017357	Pck1	-1.62833	0.021572
Alox15	2.090609	0.017466	Thbs1	-1.79951	0.021593
Vxn	1.54464	0.017604	Trim9	1.643711	0.022081
Lrrc4c	1.588237	0.017688	Tnfaip6	1.563206	0.022388
AABR07007035.1	-11.5271	0.017717	AABR07007026.1	-2.14295	0.022496
Ccl22	-2.21222	0.017846	Parm1	1.552742	0.022877
Capn6	1.820235	0.017901	Cftr	-3.86516	0.022954
AABR07014275.1	-14.3235	0.018001	LOC108348096	3.200007	0.022965
Duox1	-1.59984	0.018106	Gkn3	-7.16872	0.023152
AABR07016919.1	-2.1777	0.023362	SNORD22	1.594693	0.023229
Hp	1.554348	0.023404	Txk	-1.61811	0.030552
AABR07056767.1	-1.71358	0.024009	Pla2g7	-3.46019	0.030794
AABR07034833.2	-3.03546	0.024063	Rph3al	-1.50918	0.030805
C1qtnf3	2.281791	0.024246	Gcnt3	-1.78255	0.030837
AABR07059679.1	2.915207	0.024575	Tmem26	1.530125	0.031177
Eno3	1.706433	0.024869	Tecta	7.223467	0.031255
Cfap53	-1.61258	0.025555	Tmem108	-4.72978	0.031337
Dusp13	6.790414	0.026047	Ampd1	3.311634	0.031408
Il2rb	-1.55646	0.026119	Cd79al	-4.06453	0.031645
Slc45a3	-1.68261	0.026184	AABR07017846.1	-1.937	0.031708
AC139605.1	-10.3712	0.026273	AABR07034739.3	-4.20066	0.03204
Krt23	-1.68402	0.026755	Scd	3.187781	0.032195
LOC685351	-4.38402	0.026897	Ucp3	-1.83076	0.032291
Myom2	2.392961	0.027438	AABR07061022.2	-3.05172	0.032359
Pappa1	-2.14825	0.027629	AABR07066567.1	3.296878	0.033003
Fbp2	1.725232	0.027862	Adamts6	-1.53341	0.033066
Bcl11b	-1.63523	0.028022	Kcnk16	2.138068	0.033254
Gpr35	-1.87966	0.028288	Pvalb	2.837021	0.033372
Prr4	-24.6516	0.028484	Hoxd10	-1.50079	0.033378
AABR07030890.1	-1.79381	0.028864	Nrap	3.9939	0.033488
Hells	-13.7708	0.029091	B4galnt3	-2.52925	0.033918

Slc25a22	1.645898	0.029163	AABR07065693.3	-3.35136	0.034008
Msx2	1.693481	0.029271	Neb	1.902569	0.034017
Lrrc15	2.138118	0.029476	AABR07007905.1	-7.85407	0.034086
Etnk2	1.572299	0.030102	AABR07060394.1	-6.81057	0.034266
AABR07051532.1	-15.2994	0.030103	LOC691143	-2.19635	0.034285
Itk	-1.73301	0.030537	Rhoh	-1.66241	0.034901
Sowahb	-1.55025	0.035119	Shroom1	1.557512	0.035102
Adamts16	1.850405	0.035169	Cd79a	-3.85504	0.039248
AABR07043115.1	-2.53291	0.035392	Sh2d1a	-1.84947	0.039541
LOC689230	4.496101	0.035474	LOC103690015	-1.86472	0.040162
AABR07065789.1	-13.0563	0.035596	Aqp3	-3.35501	0.040611
Cyp24a1	1.757958	0.035671	AABR07006625.2	-3.42502	0.040803
Kng1	-3.23755	0.035732	Abcg2	-3.41617	0.040853
Fgf23	-6.50942	0.035784	Skint1	-16.3066	0.040929
Fkbp5	-1.86536	0.03581	Plcxd2	-2.01504	0.040999
Lax1	-3.191	0.036091	Tbc1d10c	-1.56933	0.041288
Col9a1	1.861571	0.036227	AABR07044346.1	-2.91179	0.04144
Fabp3	-6.23507	0.036364	LOC681385	-6.58863	0.04176
Nlrc3	-1.73019	0.036443	Agtr2	3.205947	0.041775
Ighg1	-5.76325	0.036557	Olr1	-2.96432	0.042036
AABR07065651.6	-5.43954	0.036601	Rinl	-1.55375	0.042333
AABR07065699.3	-5.43954	0.036601	Fcer2	-2.86778	0.042418
AABR07065705.5	-5.43954	0.036601	Traf3ip3	-1.53563	0.042805
AABR07051670.1	-2.81423	0.036963	Muc5b	-6.90292	0.042897
Vax2	1.567514	0.037057	Crispld1	1.561831	0.043196
Cxcl3	-7.49547	0.037198	Nim1k	1.513745	0.043391
Vstm2l	-11.0865	0.037418	Pygm	1.918239	0.044241
Cd27	-2.60405	0.037567	AABR07053613.1	-1.59369	0.044678
RGD1565617	-8.39791	0.037877	Odf4	2.374782	0.044758
Wbp11l1	-2.11035	0.038282	Cdhr1	-5.25536	0.044784
AABR07051746.1	-13.3105	0.038348	AABR07065768.1	-9.97664	0.044875
Col11a1	1.641216	0.038444	LOC103694404	9.713985	0.045198
AABR07061134.1	-3.1785	0.039025	Ms4a4c	-2.11724	0.04526
Hbegf	-1.6534	0.039223	Agr3	-5.74074	0.045505
Nrk	2.084187	0.046295	RGD1565462	-7.30606	0.045617
Ephx2	2.576515	0.046412			
Cacng1	5.495219	0.0468			
AABR07051551.1	-5.28946	0.046838			
Klhl31	2.248383	0.047183			
Sypl2	4.659486	0.048401			
Adamts19	1.857722	0.048493			
Csn1s2a	-8.36776	0.048819			
AABR07018050.1	-2.79908	0.049252			
AABR07051626.2	-4.5711	0.049254			
Fras1	1.542408	0.049506			
Scube3	-1.80761	0.049661			

SNORA73	2.679567	0.049691
Reg3b	2.049862	0.049809

Table S5. 358 differentially expressed genes between group housed rats (GH) treated with JGT and GH rats not treated with JGT. Criteria for differential expression: p-value less than 0.05, fold change (FC, between GH+JGT/GH) greater or equal to 1.5.

Gene symbol	FC	p-value	Gene symbol	FC	p-value
Ebna1bp2			Xcr1	2.367426	0.001102
Tnr	1.767593	5.8E-08	AABR07058976.1	-10.2687	0.001137
AABR07065812.2	-8.49095	3.08E-07	Mmp13	-8.96271	0.001399
S100a9	6.172351	8.77E-07	Il17b	5.963779	0.001405
Cdh17	-4.99666	1.51E-06	Mx2	-2.39524	0.001478
AABR07015066.1	3.318565	2.67E-06	Rasgrf1	-2.00885	0.001693
AABR07015078.1	-18.3363	3.36E-06	AABR07055801.1	1.688318	0.001712
AABR07015055.1	-18.3363	3.36E-06	Orm1	5.215147	0.001716
AABR07015080.2	-18.3363	3.36E-06	LOC688459	2.224153	0.001879
AABR07063424.1	-18.3363	3.36E-06	Cdkn1a	-1.79376	0.001889
AABR07015081.2	-18.3363	3.36E-06	Ppp1r3b	2.220488	0.001893
AABR07000398.1	-35.9909	5.05E-06	C3ar1	-1.5342	0.002083
AABR07070810.1	-24.9071	5.55E-06	AABR07028995.2	-1.76329	0.0025
AC128859.3	-53.8497	8.02E-06	AABR07016950.1	-7.67144	0.002683
Gad2	-1.82527	8.27E-06	AABR07004868.1	2.496433	0.00362
AABR07015057.1	22.11365	1.27E-05	Slc13a3	-8.46576	0.003643
Slfn4	-16.3341	1.75E-05	Gdap1	3.158525	0.003808
Ifit1bl	-1.92068	1.97E-05	Wap	24.20503	0.004189
AABR07051707.1	-1.99726	4.23E-05	Pcdh10	-2.43737	0.004292
Glycam1	3.821333	6.69E-05	Gpr37	-1.55295	0.004313
AC093965.1	241.9396	0.00008	Mmp8	-2.78309	0.004411
Csf3r	33.90389	9.36E-05	AABR07034362.2	-4.65343	0.004527
AABR07071765.1	-2.02541	0.000145	Syt13	1.894482	0.004642
Vil1	2.097859	0.000221	Csn2	15.9451	0.004701
Tspan8	5.897571	0.000234	Pou3f3	-2.44392	0.004981
R3hdml	1.559842	0.00024	Pdcd5	-2.02367	0.005104
Fcgr2a	-14.4882	0.000249	LOC100909548	-3.1166	0.005167
AABR07016919.1	-1.86364	0.000251	Pou2f2	-2.15201	0.005299
Scgb2a2	-6.93545	0.000492	AABR07051562.1	3.671447	0.005317
5_8S_rRNA	12.84931	0.000506	Usp2	1.805604	0.005351
Rn5-8s	-4.40892	0.000609	Tlr12	2.049535	0.005489
Lrrc25	-4.40892	0.000609	Pank3	1.576174	0.006454
Klri1	-1.82051	0.000626	AABR07052744.1	-2.2038	0.006522
Mcemp1	1.690519	0.000667	Slc25a33	1.524398	0.006804
Calb1	-2.2346	0.000748	Tmem151a	-1.85517	0.007311
LOC108351589	-3.38033	0.00077	Flt3	1.56274	0.007355
LOC681341	2.253206	0.000917	Nlrp12	-4.56457	0.0075

Fbn2	-1.64234	0.000928	AABR07000902.1	-4.02432	0.007586
Wnt7b	-1.83019	0.00095	Odam	4.186512	0.007716
S100a8	-1.52382	0.000957	Reep6	-1.61374	0.008017
Csap1	-3.04242	0.001042	Folr1	2.519552	0.008076
Creb3l1	15.544	0.008368	LOC102556148	1.73213	0.015085
Nos2	-1.98489	0.008802	Nat8f4	1.73213	0.015085
Msln	-3.72447	0.00886	AABR07012329.1	9.137473	0.015183
Gldc	-2.04925	0.009036	AABR07030563.1	1.9696	0.015417
AC141152.2	9.3835	0.009603	Mmp12	1.508238	0.01593
Prkg2	1.720845	0.009727	Cyp2d3	-8.1994	0.015982
LOC691695	-2.58916	0.00985	Grhl3	-1.84258	0.01617
Wdr54	2.812014	0.009995	Dixdc1	1.546316	0.016349
Tgfb3	1.518763	0.010064	Nrg4	1.588349	0.016563
LOC100911319	2.03816	0.010066	Sel1l3	-2.08276	0.01662
Mup4	-1.5428	0.010132	Zbp1	-1.66566	0.016939
Smlr1	-3.33804	0.010233	Mid1ip1	1.741825	0.017002
Npas2	-12.6986	0.010338	Dusp23	-1.71961	0.017014
Ptrh1	-2.34986	0.010349	Ctxn3	2.700164	0.017239
AC127140.1	1.646574	0.010434	Unc80	2.363336	0.017288
Aoah	-2.67784	0.010475	Hephl1	21.94605	0.017327
Tnmd	-1.91003	0.011002	Fyb1	-1.57937	0.01785
AABR07035796.1	1.652041	0.011544	Stag3	-1.9722	0.017965
AABR07017006.1	-1.67459	0.011779	Cldn10	5.045869	0.018173
Unc5b	-3.53235	0.01193	LOC102553785	3.671203	0.018367
Entpd3	-1.6663	0.011961	Apln	1.983558	0.018371
Mt1	2.047859	0.012321	Sectm1a	2.092961	0.018549
Tlr9	8.041654	0.012364	AABR07060588.2	-2.94346	0.018627
Slc14a1	-1.91904	0.012376	Olr1111	13.89078	0.018636
Acod1	1.639542	0.012419	Glb1l3	-3.47449	0.018745
Adamtsl2	-4.30466	0.012438	Itih4	2.487634	0.018833
Calr4	-2.66379	0.012511	Fads1	2.053061	0.018882
Il22ra2	1.964824	0.012735	AABR07014424.1	3.811738	0.019086
Prg4	3.738648	0.013013	Lhx6	-1.603	0.019403
Apobec3	-1.61024	0.01304	Shank1	3.629311	0.019484
Cxcl13	2.265211	0.013138	Penk	-1.80645	0.019747
Add2	-2.21589	0.013215	Cracr2b	2.075155	0.019842
AABR07044366.1	-2.11081	0.01322	Gzmbl2	1.905178	0.020064
AABR07055943.1	-1.6393	0.013386	Fabp3	8.366959	0.020151
LOC500035	2.063669	0.013496	Havcr2	1.674318	0.020251
AABR07061902.2	-24.221	0.013538	Gzma	1.792093	0.020434
AC096430.2	-3.31467	0.014177	Il36rn	5.373776	0.020474
Actr3b	-2.07488	0.014516	Msrb1	1.611169	0.020557
Samhd1	1.652166	0.014888	Slc13a2	2.369698	0.020613
AABR07057233.2	-1.61191	0.014896	Ttc12	1.526719	0.020722
LOC103690007	-2.21659	0.01504	Btnl7	3.921652	0.020761
Vnn3	-1.82043	0.020791	Mcpt8l3	2.117832	0.026629

Clec4a2	1.605582	0.020822	Oacyl	2.531424	0.027065
Ccl28	-2.39539	0.020906	L2hgdh	1.638915	0.027218
Shisa8	1.910246	0.021101	Gm23346	2.313277	0.027284
Mt2A	3.124035	0.021191	Slc4a1	-1.70559	0.027335
RGD1560289	5.714614	0.021316	Clec18a	3.887438	0.027367
Fam155b	-1.54793	0.021338	Rhod	1.501631	0.027429
Nrk	-1.86025	0.021476	Fam166a	1.735583	0.027435
Krt77	-1.6905	0.021614	Myh9	-1.63792	0.027464
Jph1	8.662598	0.021841	AABR07011746.1	-2.49102	0.027612
AABR07002893.1	2.109667	0.021997	Ptger2	-1.84773	0.027618
Slc5a1	2.530909	0.022101	Hdac11	1.789666	0.027704
AABR07066871.3	2.24508	0.022133	AABR07062390.1	-1.9926	0.028638
Mt-nd4l	-2.65544	0.02224	AABR07005055.1	2.061643	0.028723
Bcl2l15	-1.70034	0.022342	Piwil1	-2.74044	0.028839
Gnas	3.248525	0.022574	Cd209f	-1.56799	0.029132
Ccl12	1.552693	0.022665	Psma8	-2.20981	0.029355
LOC103691744	-3.7918	0.022845	Ikzf4	-2.1528	0.029426
AC113785.2	1.815688	0.022961	Bdh1	2.386575	0.029439
Acsl1	-2.63207	0.023037	LOC102556447	2.17975	0.029591
Cemip	1.620424	0.023048	Gfap	2.737644	0.029973
AABR07072602.1	1.923214	0.023073	Rxrg	-1.59029	0.030125
Ppial4d	-2.57821	0.02318	AABR07052523.1	1.528874	0.030166
Egr2	2.062011	0.023457	LOC100911938	4.169291	0.030331
Drc1	-1.77852	0.023662	Cyp24a1	2.284531	0.030854
Cnksr1	7.350767	0.023812	Tpbgl	-1.69406	0.030874
Tent5b	1.619434	0.023829	Kcna5	-1.83181	0.030948
Nebl	3.147543	0.023994	C17h6orf52	2.011177	0.030953
LOC100362054	1.532759	0.024025	Tjp3	1.51612	0.031106
Abcg2	5.916299	0.024225	Greb1	-1.71532	0.031396
Ebi3	2.251345	0.024285	Fcgbp	-1.96356	0.031564
LOC108351703	-2.42314	0.024288	AABR07001416.1	-4.31502	0.031619
Egr1	-4.0285	0.024634	Tgfb1	-1.79559	0.031685
Idi1	-2.06115	0.024644	AABR07013464.1	2.131411	0.031821
Il31ra	1.597893	0.025217	Klrb1c	1.626335	0.032132
Efna5	-1.68904	0.025323	Mtfp1	1.790538	0.032226
LOC108348047	-2.28233	0.025481	C2cd4c	1.615461	0.032425
Slc38a3	-1.80003	0.025612	Mt-nd4	-1.72882	0.032601
AABR07007146.1	2.32835	0.025965	Dsg1	4.057761	0.032674
Pirb	1.621614	0.026441	Hlf	1.592333	0.032832
Ldlr	-1.80908	0.026598	Gfi1b	-2.3189	0.032836
Zfp36	1.753063	0.033426	Itih3	1.532955	0.039059
Rxfp3	-1.60575	0.033502	AABR07002885.1	1.67504	0.039134
Calml3	-2.58332	0.033565	AABR07049499.1	2.01257	0.03914
Nupr1	-1.75266	0.03374	Hif3a	-2.57567	0.039422
AC120712.2	1.719997	0.034079	Slc1a2	4.267191	0.039604
Fmnl1	-1.86743	0.034228	RGD1564854	2.030611	0.03997

Slc16a14	-1.52133	0.034382	LOC498368	1.78879	0.040414
Castor2	-1.75813	0.034467	Acot5	3.140864	0.040493
Hydin	-1.50527	0.034597	Plch1	2.478101	0.040636
Fsip2	1.566174	0.034735	Pak2	2.256585	0.04124
Kcnj1	-9.08495	0.034813	AABR07038986.1	-1.52308	0.041342
Mcrip2	-9.08476	0.034925	AABR07069913.1	3.423819	0.041479
Slc44a4	1.63241	0.03493	Esco2	1.872222	0.041709
Chid1	1.675157	0.034964	Sema6b	-1.55438	0.041842
Slco4c1	1.867199	0.034984	LOC688981	-2.0739	0.042177
Prdm16	1.95489	0.035011	Gsta1	1.5022	0.04235
Slc26a7	-1.79136	0.035064	Pinlyp	3.475551	0.042352
Gjb4	3.532666	0.035153	Fam241a	1.500946	0.042648
Olr1	-4.03701	0.035311	Fer1l6	1.876461	0.042945
Ttc16	-1.99923	0.035337	Prss39	-1.885	0.043089
Aldh1a1	2.802132	0.035437	AABR07038886.1	1.607874	0.043135
Pcsk1	2.127596	0.035484	AABR07029467.2	-1.95416	0.043277
Rasgef1c	-1.50559	0.035596	Csf2rb	-1.511	0.043369
Pik3c2g	1.729684	0.035657	Actg2	1.591154	0.043557
Dio3	1.890974	0.035771	Fam89a	1.596588	0.043627
Caps2	-2.99081	0.035974	Fcgr2b	-1.53026	0.043633
Lao1	2.116385	0.036121	Gnao1	-2.00041	0.043732
LOC100911625	5.245228	0.036254	Cxcr2	-2.02212	0.043755
Siglec10	-2.82653	0.036347	Rasef	1.527594	0.043877
Ocstamp	-1.61687	0.036538	Mcpt1l4	2.717317	0.043918
LOC100360449	2.382838	0.0366	Tmem254	1.612569	0.04423
Corin	1.695101	0.036817	Ak2	1.515778	0.044271
Klhdc7a	1.744662	0.037089	Ctf2	-7.03365	0.044515
Pglyrp1	-1.50493	0.037118	RGD1310819	1.603799	0.044714
Arhgef15	-2.085	0.037198	Gng13	1.680294	0.04474
Gk	-1.61017	0.037431	Cyp1b1	-1.55435	0.044792
AABR07026137.2	1.762994	0.037559	Nkx2-1	-2.41009	0.045038
Adap1	-2.65655	0.038047	Foxi1	3.148472	0.045043
AABR07034637.1	1.531529	0.038599	Siglec1	-1.54563	0.045157
LOC100912233	-3.86859	0.038642	LOC689230	11.97272	0.045353
AC127963.2	7.516505	0.038921	LOC100359752	-2.17037	0.045519
Usp12	-8.8728	0.045712			
Slamf9	1.651477	0.045822			
Dscaml1	-1.61973	0.045835			
Chi3l1	2.195886	0.045977			
Neu2	1.538624	0.046139			
LOC100912195	2.051606	0.046147			
Sap30	1.84728	0.046183			
AABR07072449.1	1.626578	0.046277			
Glyctk	1.506721	0.046375			
AC117330.1	1.603047	0.046581			
LOC498236	1.510383	0.04659			

AC112531.2	1.853967	0.046931
Atn1	-1.84785	0.047246
RGD1563294	-1.91685	0.047284
Nat8f5	-2.99314	0.047474
Myo5c	1.72909	0.047538
AC126292.3	1.580624	0.047729
Ntrk2	-5.17513	0.048034
LOC108348157	-1.63604	0.048124
Cdhr1	5.77932	0.048528
Plpp2	-1.87798	0.04889
Bbox1	1.624019	0.048968
Cd207	4.090744	0.048983
Atp13a4	3.159957	0.049101
Mcpt1	2.358105	0.049146
Rbm20	-5.79984	0.049315
Cacna2d3	1.601145	0.049403
Serpinb11	-1.56356	0.049436
Mir6321	3.640994	0.049483
Padi2	2.715582	0.04958
	-2.33758	0.049583

Table S6. Genes in IL6/JAK/STAT3 pathway that were upregulated in socially isolated (SI) rats, compared with group-housed (GH) rats, and suppressed by JGT in SI rats.

Gene		SI vs GH	SI+JGT vs SI	Function
A2M	alpha-2-macroglobulin	0.266	-0.335	Protease inhibitor and cytokine transporter. It uses a bait-and-trap mechanism to inhibit a broad spectrum of proteases, including trypsin, thrombin and collagenase. It can also inhibit inflammatory cytokines, and it thus disrupts inflammatory cascades.
CD14	CD14 molecule	0.378	-0.383	Coreceptor for bacterial lipopolysaccharide (LPS). In concert with LBP, binds to monomeric LPS and delivers it to the LY96/TLR4 complex, thereby mediating the innate immune response to bacterial LPS
CD38	CD38 molecule	0.172	-0.290	Synthesizes the second messengers cyclic ADP-ribose and nicotinate-adenine dinucleotide phosphate, the former a second messenger for glucose-induced insulin secretion. Has cADPr hydrolase activity. Moonlights as a receptor in cells of the immune system
CSF2	colony stimulating factor 2	0.563	-1.115	Cytokine that stimulates the growth and differentiation of hematopoietic precursor cells from various lineages, including granulocytes, macrophages, eosinophils and erythrocytes
CSF2RA	colony stimulating factor 2 receptor subunit alpha	0.217	-0.320	Receptor for CSF2, low affinity for granulocyte-macrophage colony-stimulating factor (GMCSF)
CSF2RB	colony stimulating factor 2 receptor subunit beta	0.243	-0.275	High affinity receptor for interleukin-3, interleukin-5 and GMCSF
CXCL10	C-X-C motif chemokine ligand 10	0.253	-0.592	Pro-inflammatory cytokine that is involved in a wide variety of processes such as chemotaxis, differentiation, and activation of peripheral immune cells, regulation of cell growth, apoptosis and modulation of angiostatic effects
CXCL9	C-X-C motif chemokine ligand 9	0.212	-0.318	Cytokine that affects the growth, movement, or activation state of cells that participate in immune and inflammatory response
HMOX1	heme oxygenase 1	0.260	-0.745	An essential enzyme in heme catabolism
IL12RB1	interleukin 12 receptor subunit beta 1	0.757	-0.508	Functions as an interleukin receptor which binds interleukin-12 with low affinity and is involved in IL12 transduction
IL18R1	interleukin 18 receptor 1	0.611	-0.881	Responsible for the binding of the proinflammatory cytokine IL18
IL1R1	interleukin 1 receptor type 1	0.176	-0.518	Receptor for IL1A, IL1B and IL1RN. After binding to IL-1 associates with the coreceptor IL1RAP to form the high affinity IL-1 receptor complex which mediates IL-1-dependent activation of NF-kappa-B, MAPK and other pathways
IL6ST	interleukin 6 signal transducer	0.265	-0.465	A signal transducer shared by many cytokines, including interleukin 6 (IL6), ciliary neurotrophic factor (CNTF), leukemia inhibitory factor (LIF), and oncostatin M (OSM)

IRF9	interferon regulatory factor 9	0.207	-0.448	Transcription factor that plays an essential role in anti-viral immunity. It mediates signaling by type I IFNs (IFN-alpha and IFN-beta)
OSMR	oncostatin M receptor	0.352	-0.634	Associates with IL31RA to form the IL31 receptor. Binds IL31 to activate STAT3 and possibly STAT1 and STAT5.
SOCS1	suppressor of cytokine signaling 1	0.322	-0.319	Functions downstream of cytokine receptors, and takes part in a negative feedback loop to attenuate cytokine signaling. Induced by a subset of cytokines, including IL2, IL3 erythropoietin (EPO), CSF2/GM-CSF, and IFN-gamma
STAT3	signal transducer and activator of transcription 3	0.239	-0.384	Signal transducer and transcription activator that mediates cellular responses to interleukins, KITLG/SCF, LEP and other growth factors. Acts as a regulator of inflammatory response by regulating differentiation of naive CD4(+) T-cells into T-helper Th17 or regulatory T-cells (Treg)

Table S7. Genes in OXPHOS pathway that were suppressed in socially isolated (SI) rats, compared with group-housed (GH) rats, and upregulated by JGT in SI rats.

**Gene**		**SI vs GH**	**SI+JGT vs SI**	**Function/Component**
ACAT1	acetyl-CoA acetyltransferase 1	-0.299	0.397	Catalyzes the last step of the mitochondrial beta-oxidation pathway, an aerobic process breaking down fatty acids into acetyl-CoA
ACO2	aconitase 2	-0.237	0.238	Catalyzes the interconversion of citrate to isocitrate via cis-aconitate in the second step of the TCA cycle
AIFM1	apoptosis inducing factor mitochondria associated 1	-0.225	0.645	Functions both as NADH oxidoreductase and as regulator of apoptosis
ALAS1	5'-aminolevulinate synthase 1	-0.277	0.376	Encodes a mitochondrial enzyme that catalyzes the rate limiting step in heme biosynthesis
ALDH6A1	aldehyde dehydrogenase 6 family member A1	-0.419	0.310	Encodes a mitochondrial enzyme that catalyzes the irreversible oxidative decarboxylation of malonate and methylmalonate semialdehydes to acetyl- and propionyl-CoA.
ATP5F1A	ATP synthase F1 subunit alpha	-0.287	0.600	Encodes a subunit of mitochondrial ATP synthase
ATP5F1B	ATP synthase F1 subunit beta	-0.260	0.579	Encodes a subunit of mitochondrial ATP synthase
ATP5F1C	ATP synthase F1 subunit gamma	-0.289	0.508	Encodes a subunit of mitochondrial ATP synthase
ATP5F1E	ATP synthase F1 subunit epsilon	-0.277	0.536	Encodes a subunit of mitochondrial ATP synthase
ATP5MC3	ATP synthase membrane subunit c locus 3	-0.399	0.269	Encodes a subunit of mitochondrial ATP synthase
ATP5ME	ATP synthase membrane subunit e	-0.455	0.513	Encodes a subunit of mitochondrial ATP synthase
ATP5MF	ATP synthase membrane subunit f	-0.244	0.341	Encodes a subunit of mitochondrial ATP synthase
ATP5MG	ATP synthase membrane subunit g	-0.359	0.553	Encodes a subunit of mitochondrial ATP synthase
ATP5PB	ATP synthase peripheral stalk-membrane subunit b	-0.326	0.585	Encodes a subunit of mitochondrial ATP synthase
ATP5PD	ATP synthase peripheral stalk subunit d	-0.318	0.454	Encodes a subunit of mitochondrial ATP synthase
ATP5PF	ATP synthase peripheral stalk subunit F6	-0.245	0.252	Encodes a subunit of mitochondrial ATP synthase
ATP5PO	ATP synthase peripheral stalk subunit OSCP	-0.227	0.453	Encodes a subunit of mitochondrial ATP synthase
COX4I1	cytochrome c oxidase subunit 4I1	-0.209	0.195	Nuclear encoded subunit of cytochrome C oxidase
COX5B	cytochrome c oxidase subunit 5B	-0.268	0.493	Nuclear encoded subunit of cytochrome C oxidase
COX6B1	cytochrome c oxidase subunit 6B1	-0.213	0.439	Nuclear encoded subunit of cytochrome C oxidase
COX6C	cytochrome c oxidase subunit 6C	-0.301	0.306	Nuclear encoded subunit of cytochrome C oxidase
COX7A2	cytochrome c oxidase subunit 7A2	-0.228	0.538	Nuclear encoded subunit of cytochrome C oxidase
COX7A2L	cytochrome c oxidase subunit 7A2 like	-0.260	0.177	Nuclear encoded subunit of cytochrome C oxidase
COX7B	cytochrome c oxidase subunit 7B	-0.399	0.647	Nuclear encoded subunit of cytochrome C oxidase
COX7C	cytochrome c oxidase subunit 7C	-0.322	0.4563	Nuclear encoded subunit of cytochrome C oxidase
CYB5A	cytochrome b5 type A	-0.582	0.407	Encodes a protein that is a membrane bound cytochrome that reduces ferric Hb to ferous Hb
CYC1	cytochrome c1	-0.303	0.686	Encodes a subunit of cytochrome bc1 complex that plays an important role in mitochondrial respiratory chain.
CYCS	cytochrome c, somatic	-0.418	0.401	Encodes a heme protein that plays central role in electron transport chain in mitochondria
DLAT	dihydrolipoamide S-acetyltransferase	-0.503	0.420	Encodes component E2 of pyruvate dehydrogenase complex
DLD	dihydrolipoamide dehydrogenase	-0.356	0.377	Encodes a protein that functions as a dehydrogenase (as a dimer) in multi-enzyme complexes

ECHS1	enoyl-CoA hydratase, short chain 1	-0.526	0.273	Encodes a protein that functions in the second step of mitochondrial fatty acid beta-oxidation pathway
ECI1	enoyl-CoA delta isomerase 1	-0.260	0.422	Encodes a protein that is a mitochondrial enzyme involved in beta-oxidation.
ETFA	electron transfer flavoprotein subunit alpha	-0.324	0.223	Encodes protein that catalyzes initial step of mitochondrial fatty acid beta-oxidation.
ETFDH	electron transfer flavoprotein dehydrogenase	-0.187	0.312	Encodes a component of electron transfer system in mitochondria
FH	fumarate hydratase	-0.283	0.460	Encoded protein in a component of the enzyme that catalyzes formation of L-malate from fumarate
FXN	frataxin	-0.268	0.543	Encoded protein functions in regulation mitochondrial iron transport and respiration
GPX4	glutathione peroxidase 4	-0.282	0.293	Encoded protein catalyzes the reduction of hydrogen peroxide and protects against membrane lipid peroxidation
HADHB	hydroxyacyl-CoA dehydrogenase trifunctional multienzyme complex subunit beta	-0.314	0.207	Encodes a component of mitochondrial trifunctional protein, which catalyzes mitochondrial beta-oxidation of long chain fatty acids
IDH2	isocitrate dehydrogenase (NADP(+)) 2	-0.371	0.383	Encodes an enzyme that catalyzes the oxidative decarboxylation of isocitrate to 2-oxoglutatrate
IDH3B	isocitrate dehydrogenase (NAD(+)) 3 non-catalytic subunit beta	-0.343	0.539	Encoded protein is a non-catalytic component of one of the five isocitrate dehydrogenease isoenzyme
IDH3G	isocitrate dehydrogenase (NAD(+)) 3 non-catalytic subunit gamma	-0.328	0.587	Encoded protein is a subunit of isocitrate dehydrogenease enzyme complex
MAOB	monoamine oxidase B	-0.478	0.187	Encodes an enzyme that catalyzes the oxidative de-amination biogenic and xeno-biotic amines
MDH1	malate dehydrogenase 1	-0.899	0.683	Encodes a cytosolic enzyme that catalyzes reversible oxidation of malate to oxalo-acetate
MGST3	microsomal glutathione S-transferase 3	-0.362	0.170	Encodes an enzyme that catalyzes the conjugation of reduced glutathione and leukotriene A4
MPC1	mitochondrial pyruvate carrier 1	-0.829	0.688	Encodes a protein responsible for transporting pyruvate into mitochondria.
MRPL34	mitochondrial ribosomal protein L34	-0.201	0.426	Encodes a mitochondrial ribosomal 39S subunit protein
MRPL35	mitochondrial ribosomal protein L35	-0.162	0.404	Encodes a mitochondrial ribosomal 39S subunit protein
MRPS11	mitochondrial ribosomal protein S11	-0.226	0.338	Encodes a mitochondrial small ribosomal subunit protein
MRPS12	mitochondrial ribosomal protein S12	-0.211	0.422	Encodes a mitochondrial small ribosomal subunit protein
MRPS15	mitochondrial ribosomal protein S15	-0.247	0.483	Encodes a mitochondrial small ribosomal subunit protein
MRPS22	mitochondrial ribosomal protein S22	-0.165	0.644	Encodes a mitochondrial small ribosomal subunit protein
MTRF1	mitochondrial translation release factor 1	-0.404	0.561	Encodes a protein with similarity to peptide chain release factors in bacteria and yeast
MTX2	metaxin 2	-0.232	0.369	Encodes a protein that is involved in import of protein into mitochondria
NDUFA1	NADH:ubiquinone oxidoreductase subunit A1	-0.290	0.358	Encodes for an essential component of complex I of respiratory chain in the mitochondria
NDUFA3	NADH:ubiquinone oxidoreductase subunit A3	-0.243	0.630	Encodes for an accessory subunit of complex I of respiratory chain in the mitochondria
NDUFA4	NDUFA4 mitochondrial complex associated	-0.332	0.405	Encodes for an accessory subunit of complex I of respiratory chain in the mitochondria

NDUFA5	NADH:ubiquinone oxidoreductase subunit A5	-0.207	0.437	Encodes for a subunit of complex I of respiratory chain in the mitochondria
NDUFA6	NADH:ubiquinone oxidoreductase subunit A6	-0.261	0.421	Encodes for a subunit of complex I of respiratory chain in the mitochondria
NDUFA9	NADH:ubiquinone oxidoreductase subunit A9	-0.321	0.634	Encodes for a subunit of complex I of respiratory chain in the mitochondria
NDUFAB1	NADH:ubiquinone oxidoreductase subunit AB1	-0.224	0.652	Encodes for a subunit of complex I of respiratory chain in the mitochondria
NDUFB2	NADH:ubiquinone oxidoreductase subunit B2	-0.402	0.264	Encodes for a subunit of complex I of respiratory chain in the mitochondria
NDUFB3	NADH:ubiquinone oxidoreductase subunit B3	-0.312	0.287	Encodes for an accessory subunit of complex I of respiratory chain in the mitochondria
NDUFB5	NADH:ubiquinone oxidoreductase subunit B5	-0.349	0.552	Encodes for a subunit of multi-subunit complex I of respiratory chain in the mitochondria
NDUFB6	NADH:ubiquinone oxidoreductase subunit B6	-0.447	0.271	Encodes for a subunit of multi-subunit complex I of respiratory chain in the mitochondria
NDUFB7	NADH:ubiquinone oxidoreductase subunit B7	-0.154	0.605	Encodes for a subunit of multi-subunit complex I of respiratory chain in the mitochondria
NDUFB8	NADH:ubiquinone oxidoreductase subunit B8	-0.209	0.368	Encodes for a subunit of multi-subunit complex I of respiratory chain in the mitochondria
NDUFC1	NADH:ubiquinone oxidoreductase subunit C1	-0.450	0.638	Encodes for a subunit of multi-subunit complex I of respiratory chain in the mitochondria
NDUFC2	NADH:ubiquinone oxidoreductase subunit C2	-0.181	0.312	Encodes for a subunit of multi-subunit complex I of respiratory chain in the mitochondria
NDUFS1	NADH:ubiquinone oxidoreductase core subunit S1	-0.316	0.524	Encodes for a subunit of multi-subunit complex I of respiratory chain in the mitochondria
NDUFS2	NADH:ubiquinone oxidoreductase core subunit S2	-0.204	0.494	Encodes for a subunit of multi-subunit complex I of respiratory chain in the mitochondria
NDUFS3	NADH:ubiquinone oxidoreductase core subunit S3	-0.273	0.579	Encodes for a subunit of multi-subunit complex I of respiratory chain in the mitochondria
NDUFS4	NADH:ubiquinone oxidoreductase subunit S4	-0.187	0.225	Encodes for a subunit of multi-subunit complex I of respiratory chain in the mitochondria
NDUFS6	NADH:ubiquinone oxidoreductase subunit S6]	-0.213	0.179	Encodes for a subunit of multi-subunit complex I of respiratory chain in the mitochondria
NDUFS7	NADH:ubiquinone oxidoreductase core subunit S7	-0.158	0.184	Encodes for a subunit of multi-subunit complex I of respiratory chain in the mitochondria
NDUFS8	NADH:ubiquinone oxidoreductase core subunit S8	-0.183	0.424	Encodes for a subunit of multi-subunit complex I of respiratory chain in the mitochondria
NDUFV1	NADH:ubiquinone oxidoreductase core subunit V1	-0.209	0.322	Encodes for a subunit of multi-subunit complex I of respiratory chain in the mitochondria
NDUFV2	NADH:ubiquinone oxidoreductase core subunit V2	-0.157	0.455	Encodes for a subunit of multi-subunit complex I of respiratory chain in the mitochondria
OAT	ornithine aminotransferase	-0.290	0.404	Encodes the mitochondrial enzyme, which is a key enzyme in pathway that converts arginine and ornithine into glutamate and GABA
PDHA1	pyruvate dehydrogenase E1 subunit alpha 1	-0.502	0.545	Encodes an enzyme that is a component of PDH complex that converts pyruvate to acetyl-CoA and CO_2
PDHB	pyruvate dehydrogenase E1 subunit beta	-0.739	0.707	Encodes an enzyme that is a component of PDH complex that converts pyruvate to acetyl-CoA and CO_2
PDHX	pyruvate dehydrogenase complex component X	-0.294	0.551	Encodes an enzyme that is a component of PDH complex that converts pyruvate to acetyl-CoA and CO_2
PMPCA	peptidase, mitochondrial processing subunit alpha	-0.161	0.263	Encodes the alpha subunit of a proteolytic heterodimer
PRDX3	peroxiredoxin 3	-0.453	0.574	Encodes a mitochondrial protein with antioxidant function

SDHA	succinate dehydrogenase complex flavoprotein subunit A	-0.259	0.350	Encodes a catalytic subunit of succinate dehydrogenase enzyme that converts succinate to fumarate and is also a component of c omplex II of mitochondrial respiratory chain
SDHB	succinate dehydrogenase complex iron sulfur subunit B	-0.217	0.582	Encodes a subunit of succinate dehydrogenase enzyme that converts succinate to fumarate and is also a component of complex II of mitochondrial respiratory chain
SDHC	succinate dehydrogenase complex subunit C	-0.422	0.306	Encodes a subunit of succinate dehydrogenase enzyme that converts succinate to fumarate and is also a component of complex II of mitochondrial respiratory chain
SDHD	succinate dehydrogenase complex subunit D	-0.240	0.390	Encodes a subunit of succinate dehydrogenase enzyme that converts succinate to fumarate and is also a component of complex II of mitochondrial respiratory chain
SLC25A3	solute carrier family 25 member 3	-0.230	0.245	Encodes a protein that catalyzes transport of phosphate into the mitochondria
SLC25A5	solute carrier family 25 member 5	-0.227	0.703	Encodes a protein that functions as gated pore that translocates ADP from cytoplasm into mitochondria and ATP from mitochondria to cytoplasm (paralog of SLC25A6)
SLC25A6	solute carrier family 25 member 6	-0.252	0.170	Encodes a protein that functions as gated pore that translocates ADP from cytoplasm into mitochondria and ATP from mitochondria to cytoplasm
TIMM50	translocase of inner mitochondrial membrane 50	-0.1791	0.291	Encodes a subunit of the inner mitochondrial membrane translocase complex
TIMM8B	translocase of inner mitochondrial membrane 8 homolog B	-0.284	0.176	Encodes a subunit of the inner mitochondrial membrane translocase complex
UQCR10	ubiquinol-cytochrome c reductase, complex III subunit X	-0.378	0.525	Encodes a subunit of mitochondrial respiratory chain complex III
UQCR11	ubiquinol-cytochrome c reductase, complex III subunit XI	-0.301	0.176	Encodes the smallest known subunit of mitochondrial respiratory chain complex III
UQCRB	ubiquinol-cytochrome c reductase binding protein	-0.312	0.218	Encodes a subunit of mitochondrial ubiquinol-cytochrome c oxidoreductase complex (also known as complex III of mitochondrial respiratory chain)
UQCRC1	ubiquinol-cytochrome c reductase core protein 1	-0.295	0.414	Encodes a component of ubiquinol-cytochrome c oxidoreductase complex that drives oxidative phosphorylation
UQCRC2	ubiquinol-cytochrome c reductase core protein 2	-0.294	0.636	Encodes a component of ubiquinol-cytochrome c oxidoreductase complex (also known as complex III of mitochondrial respiratory chain) that drives oxidative phosphorylation
UQCRFS1	ubiquinol-cytochrome c reductase, Rieske iron-sulfur polypeptide 1	-0.270	0.425	Encodes a component of ubiquinol-cytochrome c oxidoreductase complex (also known as complex III of mitochondrial respiratory chain) that drives oxidative phosphorylation
UQCRH	ubiquinol-cytochrome c reductase hinge protein	-0.175	0.527	Encodes a component of ubiquinol-cytochrome c oxidoreductase complex (also known as complex III of mitochondrial respiratory chain) that drives oxidative phosphorylation
UQCRQ	ubiquinol-cytochrome c reductase complex III subunit VII	-0.338	0.496	Encodes a component of ubiquinol-cytochrome c oxidoreductase complex (also known as complex III of mitochondrial respiratory chain) that drives oxidative phosphorylation
